# Role of the Stem Cell-Associated Intermediate Filament Nestin in Malignant Proliferation of Non-Small Cell Lung Cancer

**DOI:** 10.1371/journal.pone.0085584

**Published:** 2014-02-03

**Authors:** Zhenguang Chen, Jiancheng Wang, Lie Cai, Beilong Zhong, Honghe Luo, Yuantao Hao, Weihua Yu, Binchao Wang, Chunhua Su, Yiyan Lei, Amos Ela Bella, Andy Peng Xiang, Tao Wang

**Affiliations:** 1 Department of Thoracic Surgery, the First Affiliated Hospital, Sun Yat-sen University, Guangzhou, Guangdong, China; 2 Department of Cardiothoracic Surgery, Huangpu Division of the First Affiliated Hospital, Sun Yat-sen University, Guangzhou, Guangdong, China; 3 Lung Cancer Research Center of Sun Yat-sen University, Guangzhou, Guangdong, China; 4 Center for Stem Cell Biology and Tissue Engineering, Sun Yat-sen University, Key Laboratory for Stem Cells and Tissue Engineering, Ministry of Education, Guangzhou, Guangdong, China; 5 Department of Rehabilitation, the First Affiliated Hospital, Sun Yat-sen University, Guangzhou, Guangdong, China; 6 Department of Thoracic Surgery, the Fifth Affiliated Hospital, Sun Yat-sen University, Zhuhai, Guangdong, China; 7 Department of Medical Statistics and Epidemiology, School of Public Health, Sun Yat-sen University, Guangzhou, Guangdong, China; 8 Guangdong Lung Cancer Institute, Guangdong General Hospital & Guangdong Academy of Medical Sciences, Guangzhou, Guangdong, China; 9 Department of Thoracic Surgery, Cancer Center, Sun Yat-sen University, Guangzhou, Guangdong, China; 10 Department of Biochemistry, Zhongshan Medical School, Sun Yat-sen University, Guangzhou, Guangdong, China; Cincinnati Children’s Hospital Medical Center, United States of America

## Abstract

**Background:**

Nestin is associated with neoplastic transformation, but the mechanisms by which nestin contributes to invasion and malignancy of lung cancer remain unknown. Considering that proliferation is necessary for malignant behavior, we investigated the mechanism of nestin action in association with the proliferative properties of non-small cell lung cancer (NSCLC).

**Methods:**

Nestin expression was examined in NSCLC specimens and cell lines. Associations with clinicopathological features, including prognosis and proliferative markers, were evaluated. Effects of nestin knockdown on proliferation and the signaling pathways involved were further investigated.

**Results:**

Nestin was expressed in most cancer specimens and all the tumor cell lines analyzed. High nestin expression in malignant tissue was associated with high Ki-67 or PCNA levels and poor patient outcomes. Conversely, knockdown of nestin expression led to significant inhibition of tumor cell proliferation, decreased colony forming ability, and cell cycle G1 arrest. Furthermore, nestin knockdown resulted in inhibition of Akt and GSK3β activation.

**Conclusions:**

Our data demonstrate that nestin expression in NSCLC cells is associated with poor prognosis of patients and tumor cell proliferation pathway. Downregulation of nestin efficiently inhibited lung cancer cell proliferation, which might be through affecting cell cycle arrest and Akt-GSK3β-Rb signaling pathway.

## Introduction

Lung cancer is now the leading cause of cancer-related deaths worldwide. In non-small cell lung cancer (NSCLC), which accounts for 80% of all lung cancer cases, distant metastases develop in up to 70% of patients with early-stage disease [1], [2]. Despite the introduction of new chemotherapeutic agents and improved surgical techniques, NSCLC remains a considerable therapeutic challenge. The survival rate is currently poor, with only 15% patient survival at 5 years after diagnosis [3]. Malignant features of NSCLC involve several important events, including proliferation and invasion of primary tumor, sustained angiogenesis, and evasion of apoptosis. Proliferation of primary tumor is an integral part of molecular and cellular pathogenesis, development, and metastasis of lung cancer [4].

Nestin, a member of the intermediate filament (IF) family, has been identified as a potential proliferative and multipotency indicator in several progenitor cells [5]–[10]. Recent reports support a link between nestin and malignant characteristics [11]–[19], and suggest that abundant nestin expression is correlated with greater malignancy and poorer prognosis in different cancers [11], [18]–[21]. However, the specific function of nestin in invasive and metastatic behavior of lung cancer cells remains unclear. The findings that nestin knockdown reduces cultured neuroblastoma and astrocytoma cell growth while its overexpression has a cytoprotective effect against H_2_O_2_ injury suggest a role in the promotion of cell survival and proliferation [22]–[24]. In contrast, another study showed that nestin downregulation does not alter the *in vitro* or *in vivo* growth characteristics of two distinct pancreatic cancer cell lines [25]. Thus, nestin may not merely act as a structural protein, but may actively participate in the control of important cellular processes. However, the precise mechanisms of nestin action in proliferation require further elucidation.

Our previous study confirmed nestin expression in NSCLC tissue samples, which appeared to correlate with the newborn lymphatic duct induced by tumor cells [21]. On the other hand, Ryuge S had described nestin expression is a prognostic indicator of poorer survival probability for patients with resected NSCLC [26]. However, the relationship between nestin expression and proliferative behavior of NSCLC cells has not been directly investigated to date. Given the limited available data on the pathophysiological role of nestin in NSCLC cells [21], [26], we have not only confirmed that the expression of nestin in NSCLC samples appeared to correlate with clinical measures of tumor malignancy, but also examined the association of nestin expression with proliferative properties of lung cancer cells and its functional role in tumor cell proliferation in the current study.

## Materials and Methods

### Tissue Specimens

A total of 71 NSCLC samples and tumor-adjacent tissues (furthest edge of resection from the tumor) were randomly selected from our tissue database. Samples were obtained from patients treated in the Department of Thoracic Surgery from the First Affiliated Hospital of Sun Yat-sen University between May 2003 and July 2004. None of the patients had received neoadjuvant chemotherapy or radiotherapy. Clinical information was obtained by reviewing preoperative and perioperative medical records or via telephone or written correspondence. Cases were staged based on the tumor-node-metastasis (TNM) classification of the International Union Against Cancer, revised in 2002 [27], [28]. The use of human materials was approved by the Medical Ethical Committee of The First Affiliated Hospital, Sun Yat-sen University (Full name of the board/committee: the Medical Ethical Committee of The First Affiliated Hospital, Sun Yat-sen University. No. 2008-7). We confirm that written informed consent from the donor or the next of kin was obtained for use of this sample in research. Clinical characteristics of patients are shown in Table 1.

**Table 1:** Association of nestin expression with clinicopathological features of NSCLC patients and primary tumors.

Characteristics		No. of patients	Nestin		*P-*value*
			Low *n* (%)	High *n* (%)	
Gender	Male	51	27 (52.9)	24 (41.1)	0.605
	Female	20	9 (45.0)	11 (57.3)	
Age (y)	≤60	40	19 (47.5)	21 (52.5)	0.634
	>60	31	17 (54.8)	14 (45.2)	
Smoking	Yes	42	19 (45.2)	23 (54.8)	0.337
	No	29	17 (58.6)	12 (41.4)	
Differentiation	Well and moderate	26	19 (73.1)	7 (26.9)	0.006
	Poor	45	17 (37.8)	28 (62.2)	
Histology	Adeno	35	9 (25.7)	26 (74.3)	0.001
	SCC	34	26 (76.5)	8 (23.5)	
	LC	2	1 (50.0)	1 (50.0)	
TNM stage	I	32	19 (59.4)	13 (40.6)	0.402
	II	17	7 (41.2)	10 (58.8)	
	III+IV	22	10 (45.5)	12 (54.5)	
Ki-67expression	High	35	9 (25.7)	26 (74.3)	0.001
	Low	36	27 (75.0)	9 (25.0)	

Abbreviations: Adeno, adenocarcinoma; SCC, squamous cell carcinoma; LC, large cell carcinoma.

* Chi-square test.

### Cell Lines

Non-small cell lung cancer cell lines, A549, H1299 and H460, were obtained from the Cell Bank of the Chinese Academy of Sciences (Shanghai, China), and cultured according to the specific Cell Bank protocol.

### Immunohistochemical Staining

The immunohistochemical procedure was similar to previously reported protocols [21], [29]. Anti-nestin (AB5922; Millipore, Temecula, CA; 1∶500 dilution) and anti-Ki-67 (sc-15402, Santa Cruz, CA, USA; 1∶200 dilution) were used as the primary antibodies.

### Plasmids

For knockdown of nestin expression, retrovirus vectors (pSM2) encoding short hairpin RNAs (shRNAs), designated shRNA1 (5′-TGC TGT TGA CAG TGA GCG AGG CAG ACA TCA TTG GTG TTA ATA GTG AAG CCA CAG ATG TAT TAA CAC CAA TGA TGT CTG CCC TGC CTA CTG CCT CGG A-3′) and shRNA2 (5′-TGC TGT TGA CAG TGA GCG CGG CTA GTC CCT GCC TGA ATA ATA GTG AAG CCA CAG ATG TAT TAT TCA GGC AGG GAC TAG CCA TGC CTA CTG CCT CGG A-3′), were purchased from Open Biosystems (Huntsville, AL, USA). These retrovirus or a construct containing a scrambled shRNA sequence (control shRNA, Open Biosystems) were stably transduced into tumor cells through antibiotic selection.

### Immunofluorescence Staining

Cells were incubated with primary anti-nestin (AB5922; Millipore, Temecula, CA; 1∶500 dilution), anti-Ki-67 (Santa Cruz; 1∶200 dilution) or anti-PCNA antibody (2586; Cell Signaling Tech, Beverly, MA; 1∶2000 dilution). Specimens were mounted with Vectashield containing Hoechst 33342, and observed under a fluorescence microscope.

### RT-PCR Analysis

Total RNA was extracted, and the following primer sequences employed: nestin, 5-GAG GAC CAG AGT ATT GTG AGA C-3 and 5-CAC AGT GGT GCT TGA GTT TC-3 (368 bp) and β-actin (internal control), 5-GTG GGG CGC CCC AGG CAC CA-3 and 5-CTC CTT AAT GTC ACG CAC GAT TTC-3 (540 bp).

### Immunoblotting Analysis

Proteins were separated, electrotransferred, blocked with a solution of TBS/0.1% Tween-20, incubated with mouse anti-nestin antibody (AB5922; Millipore, Temecula, CA; 1∶500 dilution) overnight, and detected with a horseradish peroxidase-conjugated anti-mouse secondary antibody (Cell Signaling Tech, Beverly, MA, USA). GAPDH (SC-81545; Santa Cruz, CA, USA) was used as an internal control and the Image-Pro Express (MediaCybernrtics, USA) system was used.

### Colony Formation and CCK-8 Cell Proliferation Assays

Cell colony formation was determined using crystal violet staining. Cell proliferation was assayed with the Cell Counting Kit (CCK)-8 (Dojindo, Kumamoto, Japan), and determined by measuring absorbance at 450 nm.

### Assessment of DNA Synthesis via EdU Incorporation

Cells were labeled with 5-ethynyl-2′-deoxyuridine (EdU). Nuclear incorporation was assayed by detection of Alexa Fluor 488 using the Click-iT EdU Imaging Kit (Invitrogen).

### Flow Cytometry

Following washing with PBS, fifty thousand cells were analyzed using a FACS Calibur instrument (BD Biosciences, San Jose, CA) equipped with CellQuest 3.3 software. ModFit LT 3.1 trial cell cycle analysis software was employed to determine the percentages of cells in different phases of the cell cycle.

### Expression of Cell Cycle Proteins

For immunoblotting, the following antibodies used: rabbit anti-Akt (#9272), anti-GSK3β (#9332), anti-phospho (Ser21/9)-GSK3β (#9331), anti-phospho (Ser780)-Rb (#9307), anti-phospho (Ser795)-Rb (#9301), anti-phospho (Ser870/811)-Rb (#9308), mouse anti-phospho (Ser473)-Akt (#4051) and anti-retinoblastoma (Rb) protein (#9309), as well as HRP-conjugated-anti-rabbit secondary antibody. All antibodies were purchased from Cell Signaling Technology (Beverly, MA, USA). For quantitative analyses, all data were normalized to GAPDH expression.

### Statistical Analysis

All calculations were performed using SPSS V.14.0 statistical software (Chicago, IL, USA). Spearman’s coefficient of correlation, Chi-square test, and ANOVA were applied as appropriate. Overall survival (OS) was calculated from the date of surgery until the date of last follow-up, and the association of nestin expression with OS presented as Kaplan-Meier plots. Univariate and multivariate analyses were performed using Cox proportional hazards regression to determine the prognostic effects of nestin expression and potential clinical variables on OS.

## Results

### Basic Clinical Information and Follow-up Studies

In total, 51 male and 20 female patients with NSCLC subjected to curative surgical resection were enrolled in the study. The mean age of patients was 57.6±9.8 years (range, 35 to 77 years). We examined 35 lung adenocarcinoma, 34 squamous cell carcinoma and two large cell carcinoma cases. Cases were classified as stage I (n = 32), stage II (n = 17), stage III (n = 15) and stage IV (n = 7).^27, 28^ Stage IV cases included T_1–2_ N_0_ and resected solitary brain metastasis. Patient data were analyzed after 5 years of follow-up, and information obtained for 95.8% (68 of 71) of patients. The median overall survival was 25.2±1.9 months (95% CI: 21.4–29.0 months) and mean overall survival was 24.0±2.3 months (95% CI: 19.4–28.6 months).

### Association of Nestin Expression with Poor Prognosis in NSCLC Patients

**Figure 1:**
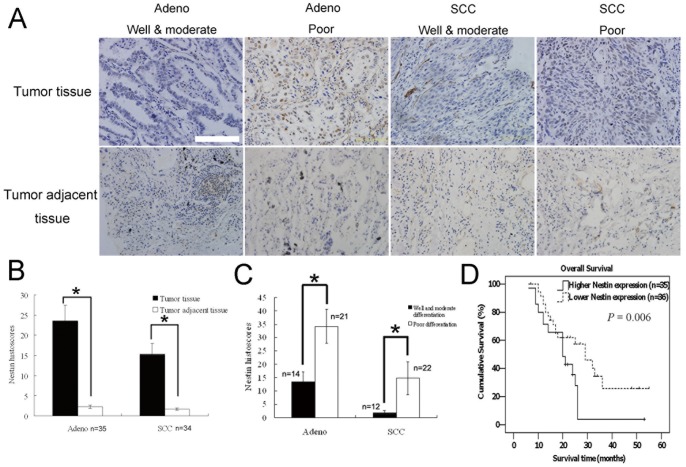
Nestin expression in NSCLC tissue and its association with prognosis of patients. Strong nestin staining was evident in NSCLC tissue, whereas staining in tumor adjacent tissue was weak (A, B). Nestin was additionally detected in poorly differentiated adenocarcinoma and squamous cell carcinoma tissue (C). A Kaplan-Meier plot depicting the differences in survival between high and low nestin expression groups, dichotomized based on the median value of nestin expression in tumor lesions (D). Scale Bar, 100 µm; *p<0.01; ANOVA.

The baseline characteristics of the study population with regard to nestin phenotype and results of multivariate analyses are presented in Tables 1 and 2, respectively. Nestin was expressed in 88.7% (63/71) cases, with almost no expression in alveolar and bronchial epithelial cells in tumor adjacent tissues (Figures 1A, B). Nestin status was correlated with poorly differentiated phenotype (*χ* ^2^ = 17.776, *P = *0.006), histological cancer tissue type (*χ* ^2^ = 8.215, *P = *0.002), N classification (*χ* ^2^ = 12.093, *P = *0.001), and vital status (*χ* ^2^ = 9.003, *P = *0.003). We observed a significant difference in survival estimates between patients with and without nestin phenotype (Figure 1D). Elevated nestin expression (using a cut-off value based on the median nestin histoscore) was associated with shorter cumulative survival (30.8±3.3 *vs* 20.2±1.7 months; hazard ratio, 3.366; *P = *0.006).

**Table 2:** Univariate and multivariate analyses of nestin expression in relation to overall survival.

Variable	Univariateanalysis		Multivariate analysis			
	HR	95% CI	*P*-value	HR	95% CI	*P*-value
Nestin-high	3.421	1.360–8.607	0.009	3.366	1.099–10.314	0.034
Nestin-low	1.000			1.000		
Adeno	1.119	0.524–2.390	0.772	2.150	0.860–5.372	0.101
SCC	1.000			1.000		
Poor	4.472	1.661–12.036	0.003	2.402	0.784–7.355	0.125
Well andmoderate	1.000			1.000		
TNM III+IV	3.276	1.907–5.627	0.001	2.358	1.329–4.186	0.003
TNM I+II	1.000			1.000		
Male	0.819	0.339–1.976	0.657	0.530	0.201–1.396	0.199
Female	1.000			1.000		
Age >60 y	0.988	0.953–1.024	0.505	1.021	0.976–1.068	0.359
Age ≤60 y	1.000			1.000		
Smoking	0.837	0.358–1.958	0.681	0.653	0.245–1.743	0.395
Non-smoking	1.000			1.000		

Abbreviations: HR, hazard ratio estimated from Cox proportional hazards regression model; CI, confidence interval of estimated HR; Adeno, adenocarcinoma; SCC, squamous cell carcinoma.

### Association of Nestin with Tumor Cell Proliferative Markers

**Figure 2:**
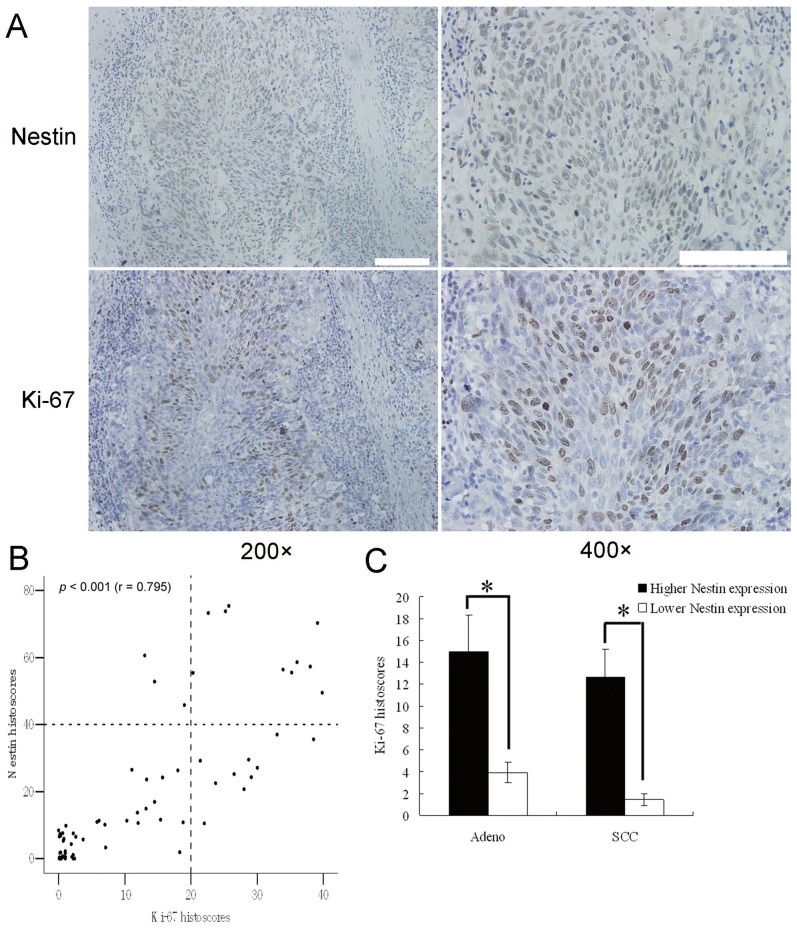
Association of nestin with tumor cell proliferative markers. Staining of nestin and Ki-67 in NSCLC specimens. (A) IHC staining of nestin and Ki-67 in NSCLC tissues. (B) Significant correlation of nestin and Ki-67 levels (r = 0.795; *P*<0.001). Each point represents one NSCLC specimen. (C) Ki-67 histoscores was elevated in adnocarcinoma (Adeno) and squamous cell carcinoma (SCC) with higher nestin expression. Scale Bar, 100 µm; *p<0.01, using ANOVA.

Nestin expression was significantly associated with those of the proliferative markers, Ki-67 (r = 0.795, *P*<0.001; Figure 2A, B, and C) and PCNA (r = 0.764, *P*<0.001; Figure S1A and B), indicative of a role in tumor cell proliferation.

### Nestin Expression in NSCLC Cell Lines

**Figure 3:**
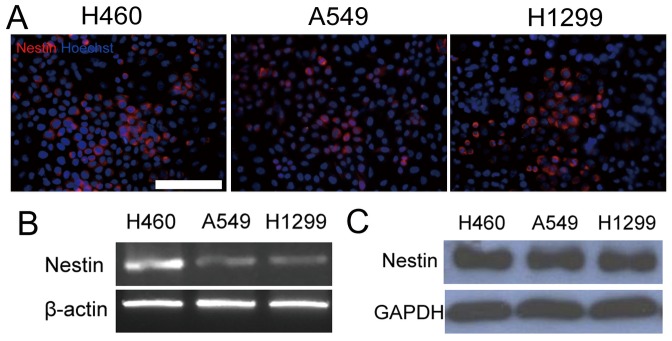
Nestin expression in NSCLC cell lines. Nestin expression was detected in A549, H1299and H460 cells using immunofluorescence staining (A), RT-PCR (B) and immunoblot analysis (C). β-actin or GAPDH was used as a loading control. Scale Bar, 100 µm.

To clarify the expression status of nestin in NSCLC, we examined its expression patterns in the NSCLC cell lines, A549 and H460. Notably, nestin mRNA and protein were detected in all cell lines (Figure 3). The expression of nestin protein between tumor cells and normal cells were also been showed using laser capture microdissection (Figure S2).

### Role of Nestin in Tumor Cell Proliferation

**Figure 4:**
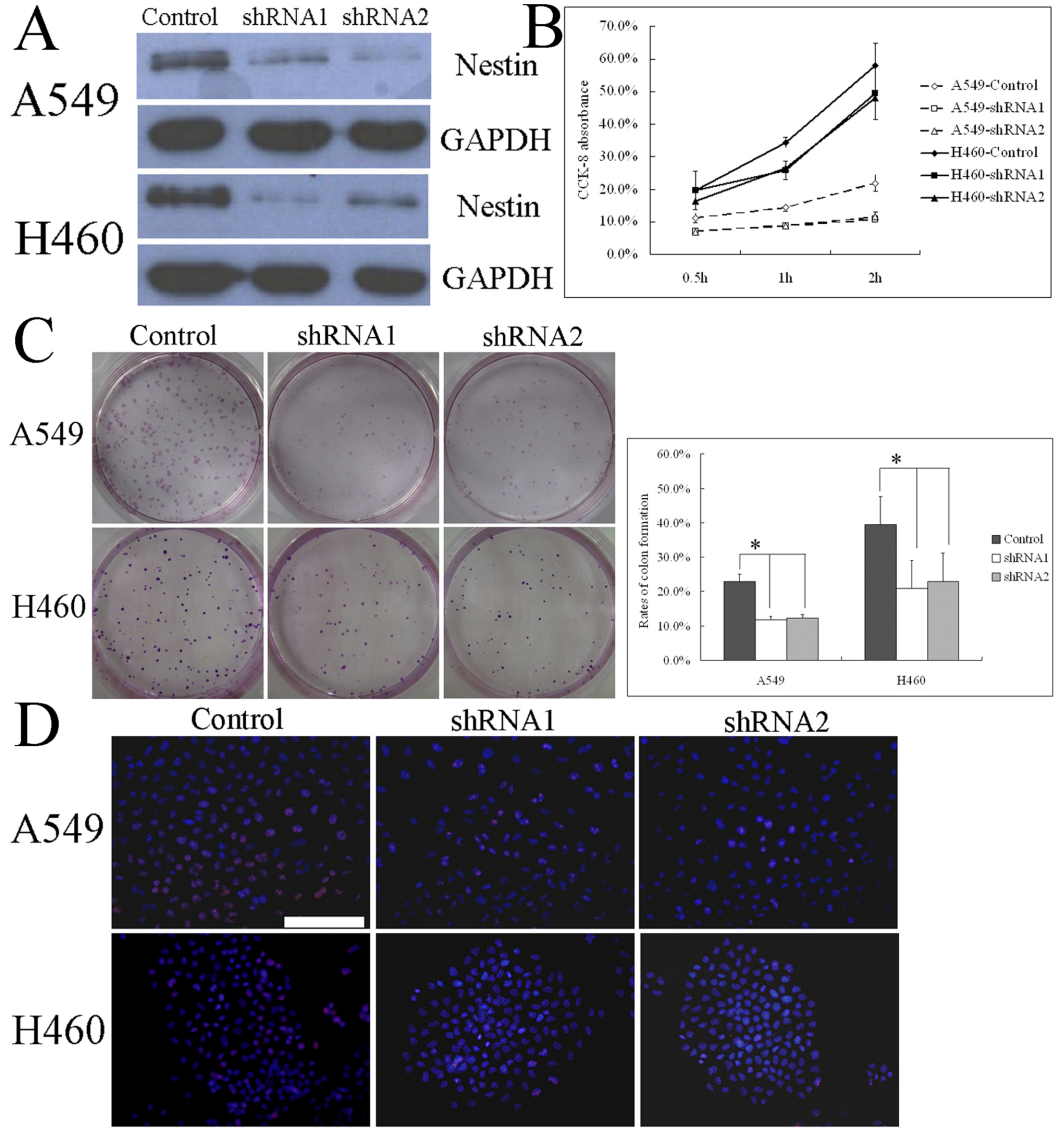
Role of nestin in tumor cell proliferation. (A) A549 or H460 cells stably expressing shRNA against nestin (shRNA1, shRNA2) were established and nestin knockdown, compared to A549 or H460 cells transfected with a scrambled shRNA sequence (control vector), confirmed with immunofluorescence staining (left panel) and immunoblotting (right panel). The differences between nestin knockdown and control A549 or H460 cells in terms of cell colony formation were demonstrated in representative micrographs with crystal violet staining (B, left panel) and quantification of cell colonies (B, right panel). Suppression of cell growth as a result of nestin knockdown was established with the CCK-8 kit assay (C). Immunofluorescence staining (D) showed positive immunoreactivity for the Ki-67 antibody (red), and nuclei were counterstained with Hoechst 33342 (blue). Scale Bar, 100 µm; *p<0.01, using ANOVA.

**Figure 5:**
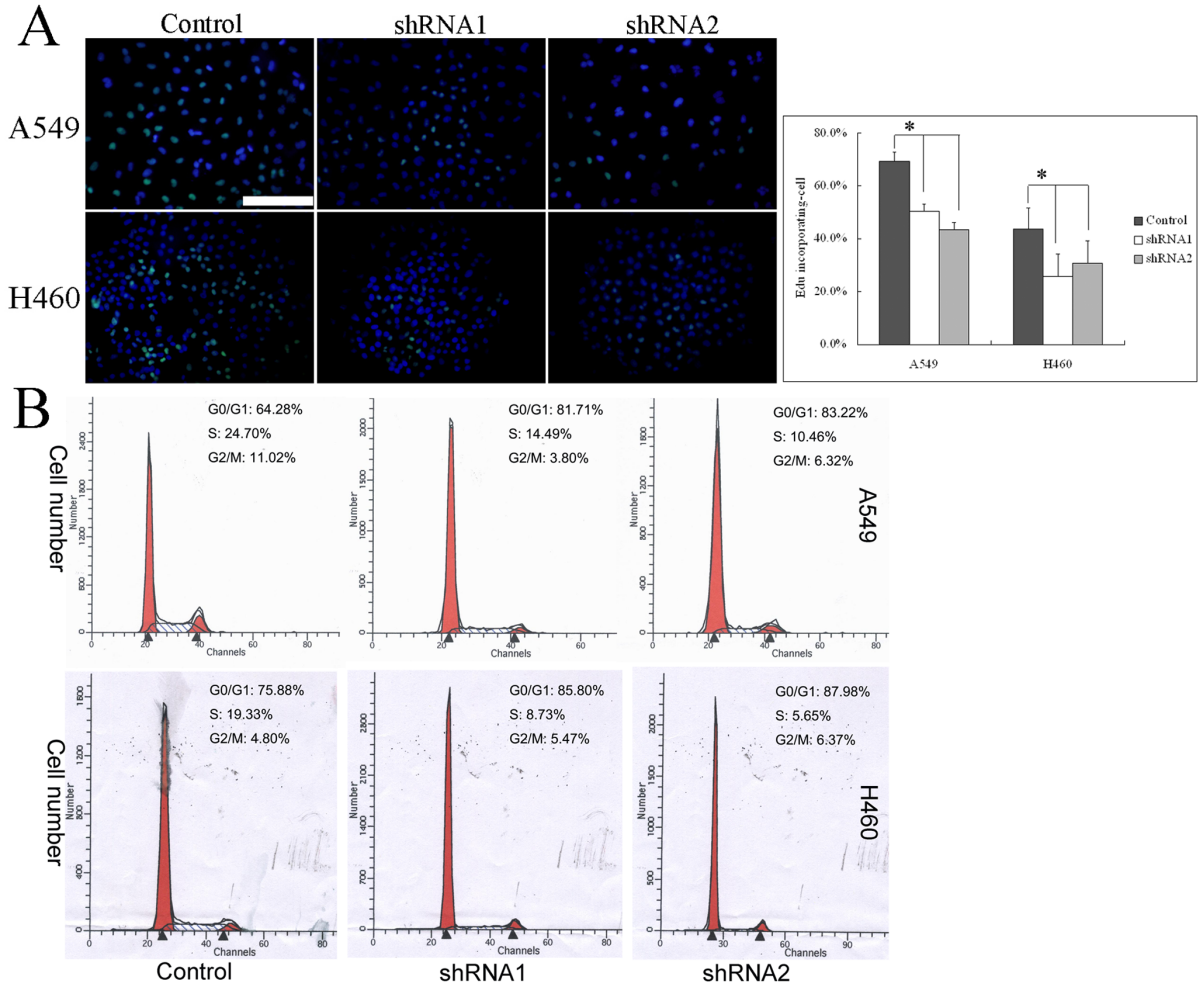
Nestin shRNA clones display DNA synthesis inhibition and cell cycle arrest. Differences in DNA synthesis rates between nestin knockdown and control A549 or H460 cells were determined using EdU incorporation assays (A). Flow cytometry was employed to analyze the cell cycle in nestin knockdown and control A549 or H460 cells (B). Scale Bar, 100 µm; *p<0.01, using ANOVA.

To assess whether nestin plays a role in lung cancer cell proliferation, we stably transfected A549 and H460 cells with plasmids encoding shRNAs targeting the nestin transcript or with scrambled shRNA control plasmids, and measured changes in proliferation-related attributes. Immunofluorescence staining and immunoblotting showed that two nestin shRNA sequences (shRNA1 and shRNA2) dramatically reduced the expression of nestin protein (Figure 4A). As shown in Figure 4B, nestin knockdown in tumor cells resulted in prominent decrease in colony-forming ability (from 23% to 11.8% and 12.3% for shRNA1 and shRNA2 in A549 cells, and from 39.6% to 20.9% and 22.8% for shRNA1 and shRNA2 in A549 cells, respectively). The proliferation rate was also markedly decreased in each nestin-knockdown cells compared to control vector cells, measured as a decrease in viable cells (Figure 4C). Consistent with the relationship between elevated nestin expression and Ki-67 or PCNA expression in NSCLC tumor specimens, nestin knockdown in tumor cells resulted in a significant decrease in the number of Ki-67-positive cells compared to control tumor cells (Figure 4D). Furthermore, DNA synthesis in nestin knockdown tumor cells was significantly inhibited compared with that in control tumor cells (Figure 5A).

### Nestin shRNA Clones Display Cell Cycle Arrest at the G1/S Phase

To determine whether tumor cell proliferative inhibition is related to cell cycle regulation, we investigated the effects of nestin knockdown on cell cycle progression in cancer cells. Notably, the number of cells in the S phase of the cell cycle was substantially reduced following nestin knockdown (Figure 5B).

### Nestin shRNA Clones Display Diminished Phosphorylation of Akt at Ser 473 and GSK3α/β at Ser 21/9

**Figure 6:**
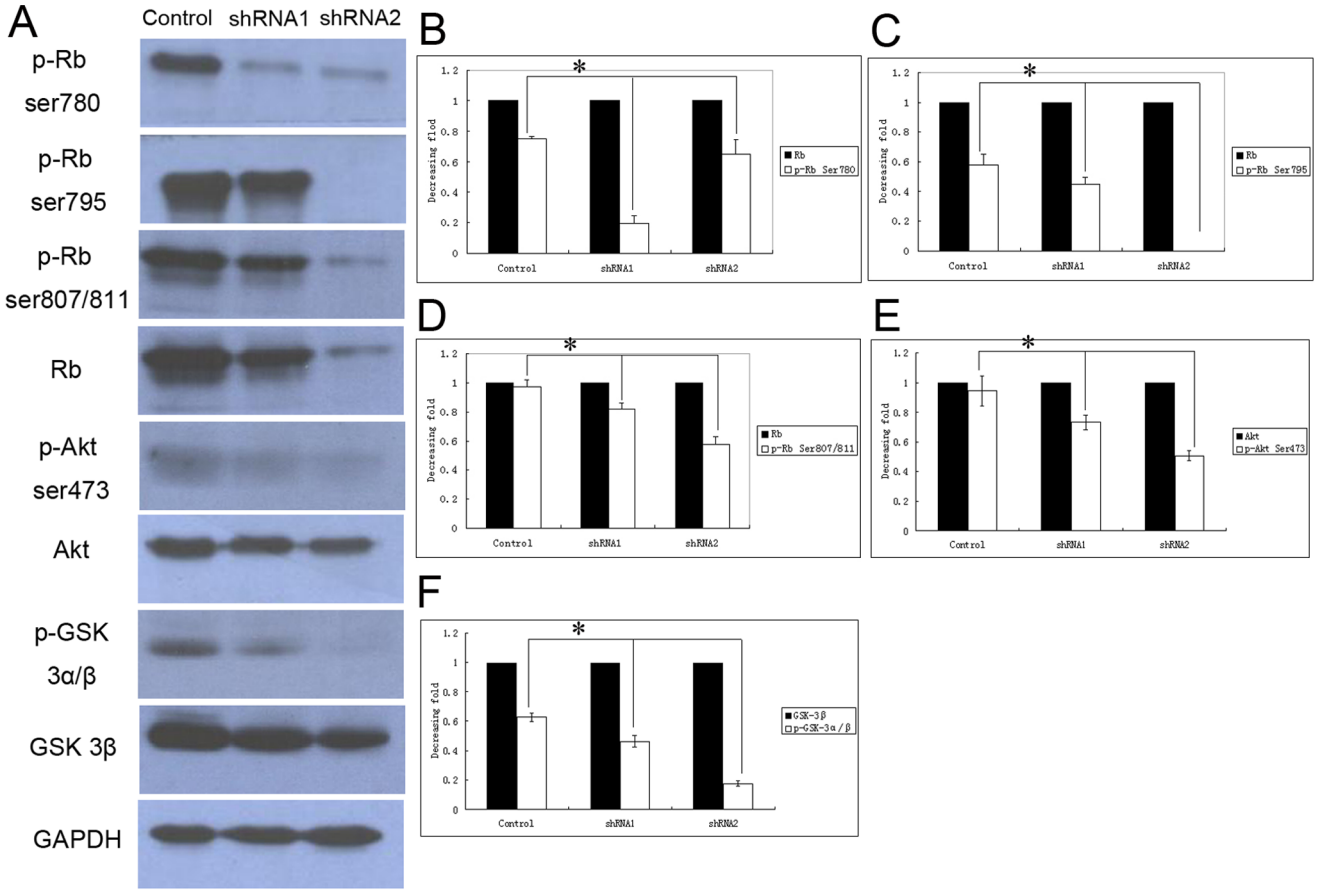
Nestin knockdown regulates Akt-GSK3β-cyclin D signaling. (A) Expression patterns of a series of cell cycle-related proteins in nestin knockdown and control A549 cells. (B–D) Quantitative analysis of Rb and phospho-Rb, (B) AKT and phospho(Ser473)-AKT (C), and GSK3β and phospho-GSK3α/β (D). *p<0.01; ANOVA.

Next, we determined the signaling profiles of nestin shRNA clones. Since the transition from the G1 to S phase is an important checkpoint during cell proliferation, we further verified the signaling pathways involved in G1 to S progression. Nestin knockdown displayed relatively diminished phosphorylation of key proteins involved in proliferation and metastasis, including Akt at Ser 473, GSK3α/β at Ser 21/9, and Rb at Ser 780, 795, and 807/811 (Figure 6A). Quantitative analysis further disclosed that the decrease in phospho-Akt and phospho-GSK3α/β proteins was particularly marked in tumor cells transduced with the shRNA2 sequence, whereas phospho-Rb expression was more significantly decreased in tumor cells transduced with the shRNA1 sequence (Figure 6B–F). Therefore, the major phosphorylation events altered upon nestin downregulation are those of the key mediators in the phosphatidylinositol 3-kinase pathway.

## Discussion

Nestin is expressed in a wide variety of embryonic and adult progenitor cell populations, and considered a marker for distinguishing precursor cells from other differentiated cells [30], [31]. Recent reports have shown that expression of nestin in breast, prostate, and pancreatic cancer is positively correlated with tumor malignancy [16]–[18]. These observations have prompted increased research interest in the expression patterns of nestin in various tumors and its relationship with proliferative and metastatic characteristics.

In the present study, we showed that nestin expression is significantly associated with malignant features of NSCLC tissue, specifically, poorly differentiated phenotype and histology classification. Additionally, nestin mRNA and protein were expressed in all the NSCLC cell lines examined. Statistical analyses revealed a significant increase in the hazard ratio (risk of dying) in patients with high nestin expression, compared with those expressing low levels of nestin. High nestin expression in NSCLC tissue was most frequently associated with disease relapse and death. Our findings further confirm previous reports of a positive relationship between nestin expression and tumor malignancy [11], [18]–[21].

Although earlier studies have reported that knockdown of nestin by siRNA effectively reduces the proliferation and growth of C6 astrocytoma cells [23] and its downregulation activates CdK5/p35-dependent apoptotic pathways [24], [32], the precise role of nestin in tumor metastasis is obscure at present. Here, we observed a positive relationship between nestin and Ki-67 expression in NSCLC tissues and cells. The observation that the Ki-67 protein is expressed during all the active phases of the cell cycle (G1, S, G2, and mitosis), but absent from resting cells, further supports the theory that nestin may be associated with tumor cell proliferation. Given the association between high nestin expression and elevated staining for the proliferative cell markers, Ki-67 and PCNA, we focused on the role of nestin in tumor cell proliferation using retroviruses to introduce shRNA vectors with nestin-targeting (or control) sequences into NSCLC cells. Significantly, proliferative properties, such as colony-forming ability and cell growth rate, were decreased in nestin shRNA clones. Our findings provide preliminary evidence for a role of nestin in lung cancer cell proliferation.

A possible mechanism to account for the link between nestin expression and proliferation is proposed on the basis of results of our analysis of cell cycle regulation in tumor cells. Specifically, the size of the S-phase cell population was dramatically decreased in nestin knockdown cells. Consistent with this finding, DNA synthesis, which occurs during the S-phase, was reduced in nestin shRNA clones. The transition from the G1 to S phase is an important checkpoint in the cell cycle. Our investigation of the key proteins that regulate this checkpoint, such as Rb protein, provided additional support for a role for nestin in proliferation via modulation of the cell cycle. Interactions of Rb with cyclin D-CDK4/6 and cyclin E-CDK2 complexes promote Rb phosphorylation and the expression of genes necessary to enter the S phase [33], [34]. Our experiments showed that nestin shRNA clones display diminished relative levels of phosphorylation at several sites of Rb. Accordingly, we propose that promotion of Rb phosphorylation is one of the molecular mechanisms through which nestin induces tumor cell proliferation. We additionally investigated specific proteins of the Akt signaling pathway. Abnormalities in the serine/threonine protein kinase, Akt, are closely related to the proliferative status of cells and induce phosphorylation of GSK3β [35], [36]. In its activated dephosphorylated form, GSK3β promotes the degradation of cyclin D, and unphosphorylated GSK3β suppresses Rb phosphorylation and prevents cell cycle progression [37], [38]. Data from the present study indicate that suppression of cell proliferation in nestin shRNA clones depends on downregulation of the Akt-GSK3β-Rb signaling pathway. By extension, these data imply that elevated nestin levels in lung cancer cells stimulate proliferation and metastasis by increasing the activity of this pathway. A number of researchers believe that nestin primarily has a cytoskeletal function, providing a reaction site for a variety of signaling pathways [39], [40], and thus cytoskeleton changes are expected to affect the kinases related to invasion and metastasis. More importantly, because A549 and H460 cell lines are p53 wild-type and p53-regulated MDM2 protein is a known regulator of Rb function, the crosstalk between nestin and p53 pathway seems to be taken into consideration, and inhibition of mTOR activity by p53, which in turn regulates phospho-Akt-S473 level, may also provide possible link between p53 and nestin [41], [42]. Hence, the link between nestin expression and regulation of Rb protein level needs to be further addressed.

Comparing with the previous reports concerning for nestin expression in lung cancer [21], [26], our findings not only confirmed that the expression of nestin in NSCLC samples appeared to correlate with clinical measures of tumor malignancy and poor histological classification, but also suggested that nestin might contribute to the proliferative and invasive behavior of NSCLC cells. In addition, our work also firstly studied the biological relationship between nestin high expression and malignant features of NSCLC using RNA knockdown techniques, cell cycle analysis, and evaluation of a series of cell cycle-associated proteins on lung cancer cell line models. Based on these results, we would provided the first demonstration that nestin phenotype exhibited enhanced proliferative properties, and explained scientifically the most important questions of the potential mechanism of nestin action in tumor cell proliferation. Thus, the phenomena that nestin-expressing tumor cells are important for proliferation, migration, and metastasis in lung cancer.

While our results indicate that nestin acts through the Akt-GSK3β-Rb signaling pathway to contribute to proliferation in lung cancer cells, other aspects of tumor malignancy, such as angiogenesis, lymphangiogenesis and tumor metabolism, are yet to be directly examined. However, in view of these findings, the role of nestin needs to be considered in ongoing clinical diagnosis or as a new target for cancer therapy.

## Conclusions

We observed nestin-positive tumor cells in the majority of NSCLC samples and significant association of nestin expression with the subset of NSCLC patients displaying poor outcomes and high levels of proliferative markers. Moreover, nestin knockdown inhibited cell proliferation and G1/S arrest in human NSCLC cells, possibly via downregulation of AKT-GSK 3β-cyclin D signaling. Our data collectively suggest that tumor cells expressing nestin promote proliferation in NSCLC, which may constitute a key mechanism of nestin-mediated malignancy in lung cancer cells. Targeted regulation of nestin may thus have therapeutic applications in human lung cancer treatment.

## Supporting Information

Figure S1 **Staining of nestin and PCNA in NSCLC specimens.** (A) IHC staining of nestin and PCNA in NSCLC tissues. (B) PCNA histoscores was elevated in adnocarcinoma (Adeno) and squamous cell carcinoma (SCC) with higher nestin expression. Scale Bar, 100 µm; *P<0.01, using ANOVA.
(TIF)

Figure S2 **The expression of nestin protein between tumor cells and normal cells using laser capture microdissection.** (A) Laser capture microdissection of tumor cells and normal cells. H&E-stained cell section showing tumor cells (a) and normal cells (d) before microdissection. Same section showing tumor cells (b) and normal cells (e) after microdissection. Tumor cells (c) and normal cells (f) following microdissection on the cap. (B) The results of western blotting of two pairs of microdissected tumor cell and normal cells. Scale Bar, 100 µm.
(TIF)

## References

[1] Jemal A, Siegel R, Ward E, Hao Y, Xu J, et al (2008) Cancer statistics, 2008. CA Cancer J Clin 58: 71–96. 18287387 10.3322/CA.2007.0010

[2] Parkin DM, Bray F, Ferlay J, Pisani P (2005) Global cancer statistics, 2002. CA Cancer J Clin 55: 74–108. 15761078 10.3322/canjclin.55.2.74

[3] D’Addario G, Felip E (2009) Non-small-cell lung cancer: ESMO clinical recommendations for diagnosis, treatment and follow-up. Ann Oncol 20 Suppl 468–70. 19454467 10.1093/annonc/mdp132

[4] Lodish H, Berk A, Kaiser CA, Krieger M, Scott MP, et al.. (2008) Molecular Cell Biology. 6th ed. New York, NY: W. H. Freeman & Company. Cancer Sections 25.1–25.5.

[5] Park D, Xiang AP, Mao FF, Zhang L, Di CG, et al (2010) Nestin is required for the proper self-renewal of neural stem cells. Stem Cells 28: 2162–2171. 20963821 10.1002/stem.541

[6] Shimizu T, Sugawara K, Tosaka M, Imai H, Hoya K, et al (2006) Nestin expression in vascular malformations: a novel marker for proliferate endothelium. Neurol Med Chir (Tokyo) 46: 111–117. 16565580 10.2176/nmc.46.111

[7] Mokry J, Cizkova D, Filip S, Ehrmann J, Osterreicher J, et al (2004) Nestin expression by newly formed human blood vessels. Stem Cells Dev 13: 658–664. 15684833 10.1089/scd.2004.13.658

[8] Amoh Y, Yang M, Li L, Reynoso J, Bouvet M, et al (2005) Nestin-linked green fluorescent protein transgenic nude mouse for imaging human tumor angiogenesis. Cancer Res 65: 5352–5357. 15958583 10.1158/0008-5472.CAN-05-0821

[9] Sejersen T, Lendahl U (1993) Transient expression of the intermediate of the intermediate filament nestin during skeletal muscle development. J Cell Sci 106: 1291–300. 8126108 10.1242/jcs.106.4.1291

[10] Li L, Mignone J, Yang M, Matic M, Penman S, et al (2003) Nestin expression in hair follicle sheath progenitor cells. Proc Natl Acad Sci USA 100: 9958–9961. 12904579 10.1073/pnas.1733025100 PMC187900

[11] Mangiola A, Lama G, Giannitelli C, De Bonis P, Anile C, et al (2007) Stem cell marker nestin and c-Jun NH2-terminal kinases in tumor and peritumor areas of glioblastoma multiforme: possible prognostic implications. Clin Cancer Res 13: 6970–6977. 18056172 10.1158/1078-0432.CCR-07-1229

[12] Bao S, Wu Q, McLendon RE, Hao Y, Shi Q, et al (2006) Glioma stem cells promote radioresistance by preferential activation of the DNA damage response. Nature 444: 756–760. 17051156 10.1038/nature05236

[13] You H, Kim YI, Im SY, Suh-Kim H, Paek SH, et al (2005) Immunohistochemical study of central neurocytoma, subependymoma, and subependymal giant cell astrocytoma. J Neurooncol 74: 1–8. 16078101 10.1007/s11060-004-2354-2

[14] Flørenes VA, Holm R, Myklebost O, Lendahl U, Fodstad O (1994) Expression of the neuroectodermal intermediate filament nestin in human melanomas. Cancer Res 54: 354–356. 8275467

[15] Brychtova S, Fiuraskova M, Hlobilková A, Brychta T, Hirnak J (2007) Nestin expression in cutaneous melanomas and melanocytic nevi. J Chtan Pathol 34: 370–375. 10.1111/j.1600-0560.2006.00627.x 17448190

[16] Li H, Cherukuri P, Li N, Cowling V, Spinella M, et al (2007) Nestin is expressed in the basal/myoepithelial layer of the mammary gland and is a selective marker of basal epithelial breast tumors. Cancer Res 67: 501–510. 17234757 10.1158/0008-5472.CAN-05-4571

[17] Kleeberger W, Bova GS, Nielsen ME, Herawi M, Chuang AY, et al (2007) Roles for the stem cell associated intermediate filament nestin in prostate cancer migration and metastasis. Cancer Res 67: 9199–9206. 17909025 10.1158/0008-5472.CAN-07-0806 PMC3072059

[18] Ohike N, Sato M, Hisayuki T, Imataka H, Sato S, et al (2007) Immunohistochemical analysis of Nestin and c-kit and their significance in pancreatic tumors. Pathol Int 57: 589–593. 17685930 10.1111/j.1440-1827.2007.02143.x

[19] Rani SB, Mahadevan A, Anilkumar SR, Raju TR, Shankar SK (2006) Expression of nestin - a stem cell associated intermediate filament in human CNS tumors. Indian J Med Res 124: 269–280. 17085830

[20] Klein WM, Wu BP, Zhao S, Wu H, Klein-Szanto AJ, et al (2007) Increased expression of stem cell markers in malignant melanoma. Mod Pathol 20: 102–107. 17143262 10.1038/modpathol.3800720

[21] Chen Z, Wang T, Luo H, Lai Y, Yang X, et al (2010) Expression of nestin in lymph node metastasis and lymphangiogenesis in non-small cell lung cancer patients. Hum Pathol 41: 737–744. 20132963 10.1016/j.humpath.2009.10.018

[22] Thomas SK, Messam CA, Spengler BA, Biedler JL, Ross RA (2004) Nestin is a potential mediator of malignancy in human neuroblastoma cells. J Biol Chem 279: 27994–27999. 15117961 10.1074/jbc.M312663200

[23] Wei LC, Shi M, Cao R, Chen LW, Chan YS (2008) Nestin small interfering RNA (siRNA) reduces cell growth in cultured astrocytoma cells. Brain Res 1196: 103–112. 18234160 10.1016/j.brainres.2007.11.026

[24] Sahlgren CM, Pallari HM, He T, Chou YH, Goldman RD, et al (2006) A nestin scaffold links Cdk5/p35 signaling to oxidant-induced cell death. EMBO J 25: 4808–4819. 17036052 10.1038/sj.emboj.7601366 PMC1618100

[25] Matsuda Y, Naito Z, Kawahara K, Nakazawa N, Korc M, et al (2011) Nestin is a novel target for suppressing pancreatic cancer cell migration, invasion and metastasis. Cancer Biol Ther 11(5): 1–12. 21258211 10.4161/cbt.11.5.14673 PMC3230315

[26] Ryuge S, Sato Y, Wang GQ, Matsumoto T, Jiang SX, et al (2011) Prognostic significance of nestin expression in resected non-small cell lung cancer. Chest 139(4): 862–869. 20829334 10.1378/chest.10-1121

[27] Goldstraw P, Crowley J, Chansky K, Giroux DJ, Groome PA, et al (2007) The IASLC Lung Cancer Staging Project: proposals for the revision of the TNM stage groupings in the forthcoming (seventh) edition of the TNM classification of malignant tumours. J Thorac Oncol 2(8): 706–714. 17762336 10.1097/JTO.0b013e31812f3c1a

[28] Groome PA, Bolejack V, Crowley JJ, Kennedy C, Krasnik M, et al (2007) The IASLC Lung Cancer Staging Project: validation of the proposals for revision of the T, N, and M descriptors and consequent stage groupings in the forthcoming (seventh) edition of the TNM classification of malignant tumours. J Thorac Oncol 2(8): 694–705. 17762335 10.1097/JTO.0b013e31812d05d5

[29] Yang XH, Wu QL, Yu XB, Xu CX, Ma BF, et al (2008) Nestin expression in different tumours and itsrelevance to malignant grade. J Clin Pathol 61: 467–473. 17873113 10.1136/jcp.2007.047605

[30] Wiese C, Rolletschek A, Kania G, Blyszczuk P, Tarasov KV, et al (2004) Nestin expression: a property of multi-lineage progenitor cells? Cell Mol Life Sci 61: 2510–2522. 15526158 10.1007/s00018-004-4144-6 PMC11924557

[31] Suzuki S, Namiki J, Shibata S, Mastuzaki Y, Okano H (2010) The neural stem/progenitor cell marker nestin is expressed in proliferative endothelial cells, but not in mature vasculature. J Histochem Cytochem 58: 721–730. 20421592 10.1369/jhc.2010.955609 PMC2907277

[32] Sahlgren CM, Mikhailov A, Vaittinen S, Pallari HM, Kalimo H, et al (2003) Cdk5 regulates the organization of nestin and its association with p35. Mol Cell Biol 23: 5090–5106. 12832492 10.1128/MCB.23.14.5090-5106.2003 PMC162223

[33] Yan S, Wenner CE (2001) Modulation of cyclin D1 and its signaling components by the phorbolester TPA and the tyrosine phosphatase inhibitor vanadate. Cell Physiol 186: 338–349. 10.1002/1097-4652(2000)9999:9999<000::AID-JCP1032>3.0.CO;2-B 11169972

[34] Manning BD, Cantley LC (2007) AKT/PKB signaling: navigation downstream. Cell 129: 1267–1274. 10.1016/j.cell.2007.06.009 PMC2756685 17604717

[35] Ahmed NN, Grimes HL, Bellacosa A, Chan TO, Tsichlis PN (1997) Transduction of interleukin-2 antiapoptotic and proliferative signals via AKT protein kinase. Proc Natl Acad Sci USA 94: 3627–3632. 9108028 10.1073/pnas.94.8.3627 PMC20491

[36] Diehl JA, Cheng M, Roussel MF, Sherr CJ (1998) Glycogen synthase kinase-3β regulates cyclin D1 proteolysis and subcellular localization. Genes Dev 12: 3499–3511. 9832503 10.1101/gad.12.22.3499 PMC317244

[37] Etienne-Manneville S, Hall A (2003) Cdc42 regulates GSK-3beta and adenomatous polyposis coli to control cell polarity. Nature 421: 753–756. 12610628 10.1038/nature01423

[38] Turjanski AG, Vaqué JP, Gutkind JS (2007) MAP kinases and the control of nuclear events. Oncogene 26: 3240–3253. 17496919 10.1038/sj.onc.1210415

[39] Pallari HM, Eriksson JE (2006) Intermediate Filaments as Signaling Platforms. Sci STKE 2006(366): pe53. 17179489 10.1126/stke.3662006pe53

[40] Coulombe PA, Wong P (2004) Cytoplasmic intermediate filaments releaved as dynamic and multipurpose scaffolds. Nat Cell Biol 6: 699–706. 15303099 10.1038/ncb0804-699

[41] Yang H, He LL, Kruk P, Nicosia SV, Cheng JQ (2006) Aurora-A induces cell survival and chemoresistance by activation of Akt through a p53-dependent manner in ovarian cancer cells. Int J Cancer 119(10): 2304–2312. 16894566 10.1002/ijc.22154

[42] Breuleux M, Klopfenstein M, Stephan C, Doughty CA, Barys L, et al (2009) Increased AKT S473 phosphorylation after mTORC1 inhibition is rictor dependent and does not predict tumor cell response to PI3K/mTOR inhibition. Mol Cancer Ther 8(4): 742–753. 19372546 10.1158/1535-7163.MCT-08-0668 PMC3440776

