# Supplementary figures and images for: Role of the Stem Cell-Associated Intermediate Filament Nestin in Malignant Proliferation of Non-Small Cell Lung Cancer
Source: PLoS One. 2014 Feb 3;9(2):e85584. doi: 10.1371/journal.pone.0085584 (PMC3911905; doi:10.1371/journal.pone.0085584)

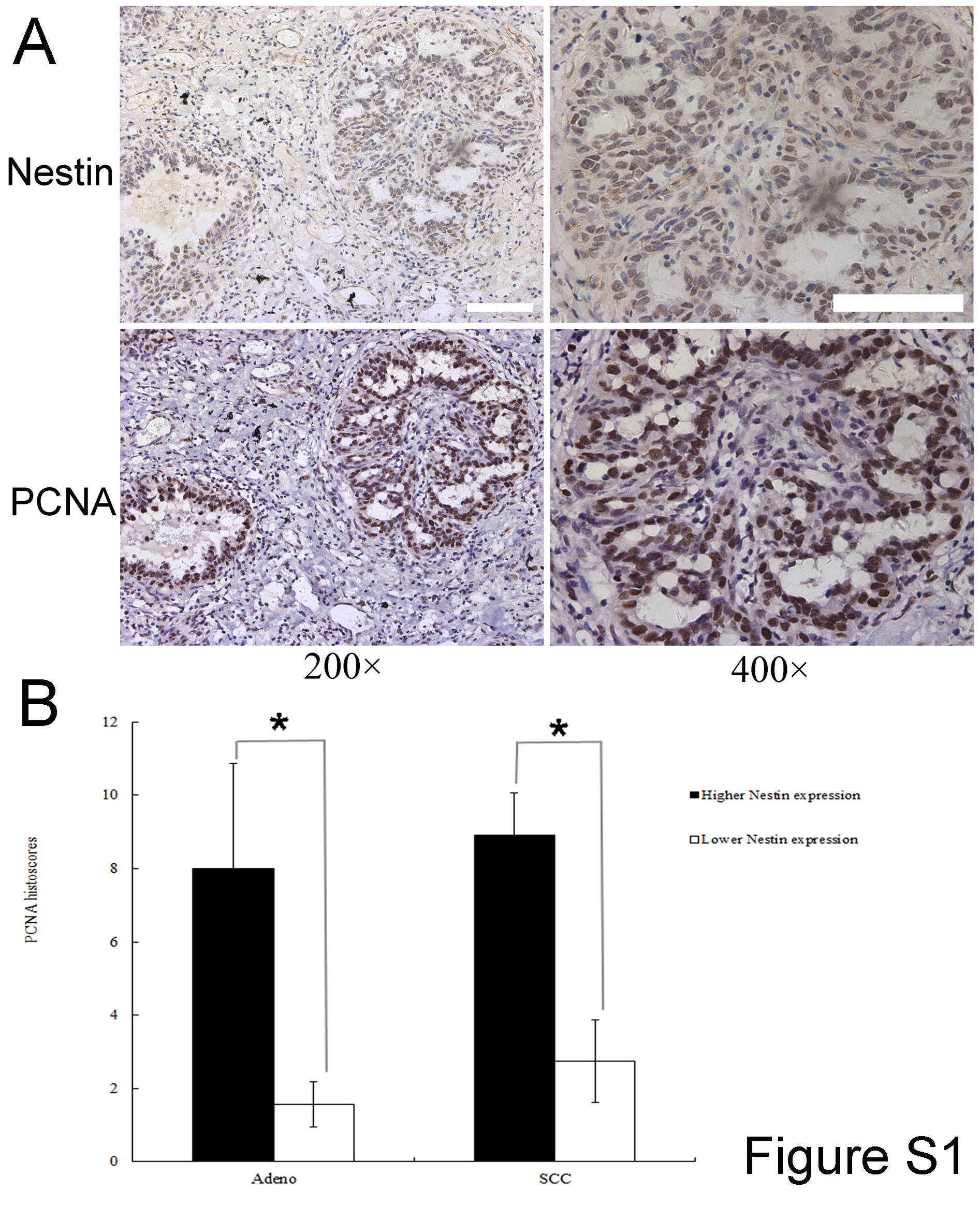

Supplement: Figure S1 — Staining of nestin and PCNA in NSCLC specimens. (A) IHC staining of nestin and PCNA in NSCLC tissues. (B) PCNA histoscores was elevated in adnocarcinoma (Adeno) and squamous cell carcinoma (SCC) with higher nestin expression. Scale Bar, 100 µm; *P<0.01, using ANOVA. (TIF) [file pone.0085584.s001.tif]

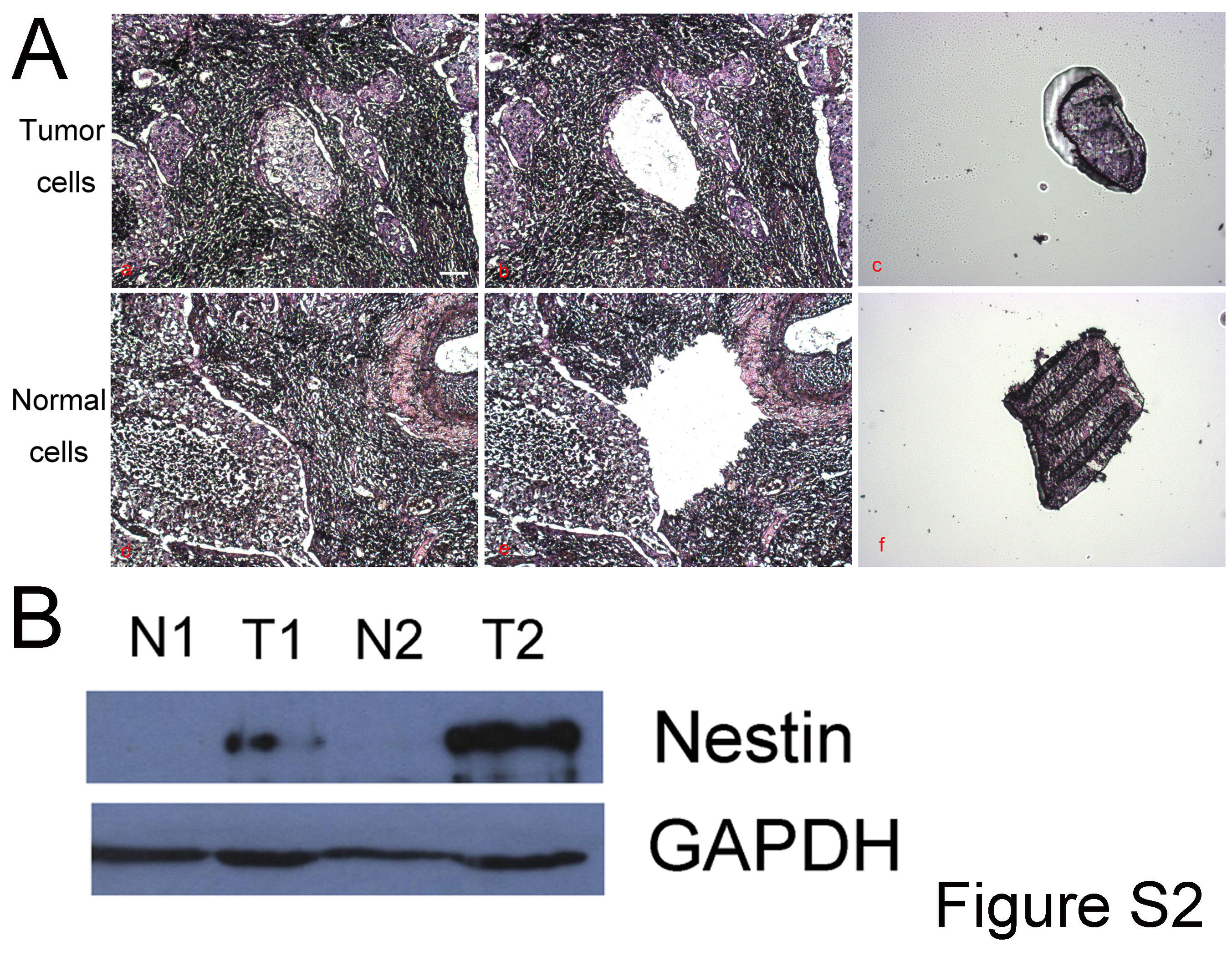

Supplement: Figure S2 — The expression of nestin protein between tumor cells and normal cells using laser capture microdissection. (A) Laser capture microdissection of tumor cells and normal cells. H&E-stained cell section showing tumor cells (a) and normal cells (d) before microdissection. Same section showing tumor cells (b) and normal cells (e) after microdissection. Tumor cells (c) and normal cells (f) following microdissection on the cap. (B) The results of western blotting of two pairs of microdissected tumor cell and normal cells. Scale Bar, 100 µm. (TIF) [file pone.0085584.s002.tif]
